# Examining the influence of user experience on functional value, satisfaction, and usage intention in OTT video platforms

**DOI:** 10.1371/journal.pone.0350557

**Published:** 2026-05-29

**Authors:** Ya-Qin You, Han-Han Song, Yu-Liang Feng

**Affiliations:** 1 School of Education, Jiangsu University of Technology, JiangSu, China; 2 Faculty of Innovation and Design, City University of Macau, Macau, China; 3 Artificial Intelligence Innovation Design Research Center, Jiangsu University of Technology, JiangSu, China; University of Naples Federico II: Universita degli Studi di Napoli Federico II, ITALY

## Abstract

With the widespread availability of high-speed networks and smart devices, OTT video platforms have become a primary channel for entertainment and information. This study investigates how user experience (UX) dimensions—convenience/fluency, interaction, emotional experience, and service-quality experience—shape functional value, satisfaction, and usage intention in OTT video platforms. Based on survey data from 230 users (21 items measured on seven-point Likert scales), a PLS-SEM analysis was conducted using SmartPLS 3 with 5,000 bootstrap resamples. The model demonstrated acceptable fit (SRMR = 0.075) and acceptable predictive performance, with R² values of 0.522 for functional value, 0.760 for satisfaction, and 0.339 for usage intention, and Q² values indicating moderate-to-high predictive relevance. Results show that convenience/fluency positively influences functional value, satisfaction, and usage intention. Interaction exerts a direct positive effect on usage intention but not on functional value or satisfaction. Emotional experience enhances functional value, satisfaction, and usage intention, with satisfaction partially mediating its effect on intention. Service-quality experience positively influences functional value and satisfaction but does not show a significant direct effect on usage intention. Its association with usage intention appears to operate indirectly through evaluative pathways. Furthermore, functional value positively influences satisfaction but does not show a significant direct effect on usage intention, whereas satisfaction serves as a significant predictor of usage intention. These findings clarify the UX-driven pathways underlying the adoption of OTT video platforms and explain why service quality and functional value alone may not translate into usage intention without satisfaction. From a practical perspective, OTT platforms should prioritize seamless performance, emotionally engaging content, and interactive features, while maintaining reliable service delivery as a basic condition for strengthening functional value and satisfaction.

## Research Background

Over-the-top (OTT) video platforms have swiftly become leading global channels for entertainment and information, fueled by advancements in network technology and mobile devices. The proliferation of high-speed networks and smart devices has enabled robust online video transmission and consumption. Recent industry reports indicate the rapid expansion of digital video consumption in China and globally, underscoring the growing importance of OTT video platforms in contemporary media environments [1,2, iSPOT Digital 3]. These indicators underscore OTT platforms’ prominence and growing potential within the new media landscape.

Research identifies relaxation, cost, convenience, content availability, and user experience as key drivers of OTT platform adoption, while content quality, interactivity, trust, and values drive user satisfaction [4,5]. Empirical findings link navigability, improved viewing experience, entertainment, relaxation, and platform interactivity to increased cord-cutting intentions [6], with performance expectancy and motivating factors further shaping usage intention [7,8]. Mulla’s [9] review summarizes over 10 influential factors, including content, price, convenience, entertainment, and socialization.

Further investigations have revealed the multidimensional nature of OTT user behavior. Singh et al. [10] found that convenience value and hedonic experience are critical drivers of continuous use in live streaming services, even leading to addictive behavior. Kim et al. [11] reported, through a cross-cultural comparison, that Chinese users prioritize resolution quality, whereas Korean users place greater emphasis on recommendation systems. Chang et al. [12] proposed the Theory of Streaming Service Acceptance (TSSA), highlighting the importance of attitude, social norms, and perceived behavioral control in predicting subscription intention for music streaming services. Kareem et al. [13], drawing on the ECM model, found that perceived usefulness and expectation confirmation significantly influence continuous subscription intention via satisfaction, with habit and content availability serving as moderators. Menon [14], based on the uses and gratifications theory, identified entertainment, social interaction, and information-seeking motivations as significant predictors of satisfaction and cord-cutting intention. Collectively, these findings indicate that research on OTT platforms has covered adoption, continuance intention, willingness to pay, user motivations, and cross-cultural differences, showing that user decisions are shaped not only by functional value but also by psychological, social, and cultural factors.

However, although prior studies have generated valuable insights into OTT platform adoption and continuance, the existing literature still shows two limitations that are relevant to the present study. First, user experience in OTT settings is often examined in a partial manner. Some studies emphasize convenience, content quality, or system performance, whereas others focus on entertainment, interactivity, or satisfaction. This body of work confirms that multiple experiential factors matter, but it does not yet provide a sufficiently integrated account of how different UX dimensions operate together within a single explanatory model.

Second, the relationship between experience and subsequent evaluation remains theoretically under-specified. Existing studies generally demonstrate that specific platform attributes are associated with satisfaction or intention, yet they less often distinguish the intermediate psychological processes through which these effects occur. In particular, the literature provides limited explanation of how experiential cues such as convenience, interaction, emotion, and service quality are transformed into cognitive evaluation and affective evaluation, and how these evaluations subsequently shape usage intention. This issue is especially relevant in OTT contexts, where user judgment may involve both instrumental appraisal and experiential value assessment [15].

To address these limitations, this study adopts the Stimulus-Organism-Response (S-O-R) perspective as an explanatory framework rather than merely a classificatory one. In the present model, multidimensional UX attributes of OTT platforms are treated as external stimuli; these stimuli are expected to influence users’ internal states in two distinguishable forms, namely cognitive appraisal of functional value and affective evaluation of satisfaction; these internal states then shape the behavioral response of usage intention [16–18]. By specifying these sequential linkages, the study seeks to clarify not only whether UX matters, but also how different experiential dimensions are translated into user intention through distinct evaluative pathways.

## Literature review

### OTT video platforms

OTT video platforms refer to Internet-based media services that deliver video content directly to users, bypassing traditional distribution channels such as broadcast and cable systems. Prior review studies have emphasized that OTT services are defined not only by their Internet-based delivery mode, but also by their support for on-demand access, flexible consumption across devices and contexts, and the growing importance of platform-level features such as navigability, interactivity, and content-related experience. Compared with traditional broadcast media, OTT platforms provide users with greater temporal and spatial flexibility, allowing them to access content according to their own schedules and viewing situations. At the same time, recent OTT research has shown that platform use is shaped not only by technical accessibility and convenience, but also by personalization, content diversity, and broader user-experience factors. These characteristics make OTT platforms an appropriate context for examining how multidimensional UX attributes are translated into perceived value, satisfaction, and continued usage intention [9,19–21].

### User Experience

User Experience (UX) originated in the field of human–computer interaction. Dong, Wu, and Dai [22] defined UX as the subjective perception formed by users during interaction with a product. Building on this, Pine and Gilmore [23] introduced the concept of the “experience economy,” arguing that experience has independent value and even surpasses that of traditional goods and services. They further developed an experience model encompassing four dimensions: educational, entertainment, esthetic, and escapist experiences. Subsequently, Schmitt [24] proposed five experiential modules—sensory, emotional, cognitive, behavioral, and relational experiences—laying a theoretical foundation for multidimensional UX research.

Empirical studies have investigated the influence of UX on satisfaction and behavioral intention across various contexts. For example, Lin [25] found that behavioral and cognitive experiences significantly affected customer satisfaction among sports protective gear consumers in the Greater Taipei area. Wu [26] demonstrated in the context of landscape restaurants that experiential marketing and experiential value were positively associated with satisfaction. Tsai and Chen [27] revealed in their study of the Chimei Museum that sensory and emotional experiences exerted the strongest effects on customer satisfaction, while relational experience played a relatively minor role. Similarly, Chang [28] showed in the department store retail sector that behavioral experience had a significant impact on satisfaction.

In the context of digital media and short video platforms, recent studies further validate the importance of UX for usage intention. Wang et al. [29] reported that perceived ease-of-use experience significantly enhanced users’ usage intention. Qi et al. [5] proposed a satisfaction evaluation framework for short video platforms based on grounded theory and the CRITIC-VIKOR method, finding that improvements in UX, content and interactivity, advertising, and privacy security effectively increased user satisfaction.

Overall, UX has emerged as a critical antecedent influencing both user satisfaction and usage intention. Its multidimensional nature and empirical evidence across different contexts provide a solid theoretical foundation for investigating the mechanisms by which UX affects usage intention on OTT video platforms.

### S-O-R theory section

The Stimulus-Organism-Response (S-O-R) paradigm, initially conceptualized by Mehrabian and Russell [16], offers a robust theoretical lens for elucidating how environmental cues drive individual behavior. This model postulates a sequential process wherein external environmental inputs (Stimuli) induce internal cognitive and affective states (Organism), which subsequently manifest in specific behavioral outcomes (Response) [17,18]. Within this framework, Stimuli refer to the external interface or atmosphere; the Organism denotes the internal processing of the user, encompassing their emotions and cognitive evaluations; and the Response represents the resulting approach or avoidance actions. The S-O-R framework has been successfully adapted across various domains, including studies on user resistance [30] and online purchasing behavior [31]. In the present study, multidimensional user experience (UX)—including convenience, interaction, emotional experience, and service quality—is conceptualized as the stimulus component. These stimuli shape two organism states, namely perceived functional value and satisfaction, which in turn influence the response of usage intention.

### Research Hypotheses

**Fig 1:**
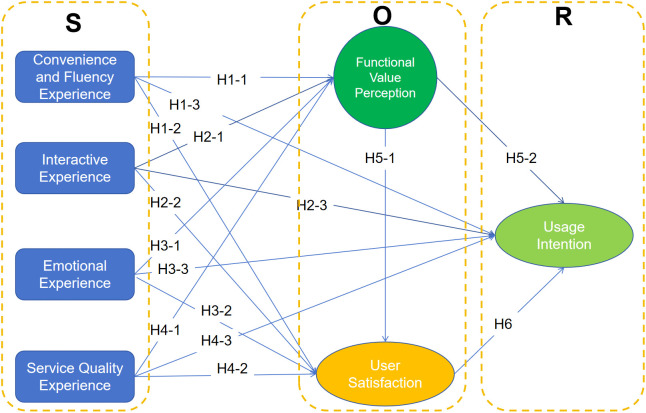
Research Hypotheses.

To move beyond a descriptive listing of constructs, we develop the hypotheses from an integrated Stimulus-Organism-Response (S-O-R) logic [16,17] combined with (a) service quality and information-systems success arguments for utilitarian evaluations [32,33], and (b) cognitive appraisal and media engagement perspectives for affective responses [34,35]. In this framework, UX attributes of an OTT platform constitute environmental stimuli (S) that shape users’ internal states (O) in two complementary ways: (1) a cognitive appraisal of instrumental utility (perceived functional value), and (2) an affective evaluation of the overall experience (satisfaction). These organism states, in turn, drive approach behavior (R), operationalized as usage intention. Fig 1 presents the research model and hypothesized relationships in this study.

Importantly, prior findings are not fully consistent: some studies indicate that interactive features increase perceived value and satisfaction, whereas others suggest that interaction primarily strengthens engagement and social attachment without necessarily improving utilitarian value judgments. This theoretical tension is consistent with dual-process views in media use, where interactive affordances can work via both utilitarian appraisal and engagement/relatedness routes [35,36]. Accordingly, we retain interaction–value/satisfaction hypotheses for theory testing, while explicitly acknowledging that these paths may be smaller or more context-dependent than those of convenience/fluency and emotional experience.

Convenience and fluency experience refers to users’ perceptions of operational smoothness and access efficiency, including cross-device convenience, interface clarity, playback smoothness, update timeliness, and perceived viewing presentation quality. From a cognitive evaluation perspective, low effort and high performance reduce perceived “costs” and increase perceived usefulness/utility [37,38], thereby enhancing functional value; simultaneously, a frictionless process improves affective evaluation, increasing satisfaction [39,40]. Consistent with this logic, facilitating conditions and seamless usability have been linked to value perceptions, satisfaction, and continuance intentions in digital services.

H1-1: Convenience and fluency experience positively influences users’ perceptions of functional value in OTT video platforms.

H1-2: Convenience and fluency experience positively influences users’ satisfaction in OTT video platforms.

H1-3: Convenience and fluency experience positively influences users’ usage intention in OTT video platforms.

Interactive experience captures the extent to which users can express opinions, interact with others, and receive feedback (e.g., likes and shares). In OTT contexts, interaction may serve dual roles. On the one hand, it can provide informational support, facilitate expression, and enhance users’ sense of participation, thereby contributing to platform evaluation. On the other hand, interaction may also strengthen engagement and social involvement without necessarily increasing utilitarian value judgments to the same degree [35,36]. This dual-route logic helps explain why the effects of interaction may differ from those of convenience and service quality, and it justifies testing both organism pathways and a direct response pathway.

H2-1: Interactive experience positively influences users’ perceptions of functional value in OTT video platforms.

H2-2: Interactive experience positively influences users’ satisfaction in OTT video platforms.

H2-3: Interactive experience positively influences users’ usage intention in OTT video platforms.

Emotional experience represents the extent to which content consumption evokes positive affect (e.g., joy and happiness). Cognitive appraisal theories argue that affective responses are outcomes of appraisals of goal congruence and meaning [34,41]; in entertainment services, positive affect also acts as a “value signal” that users integrate into overall evaluations [15]. Therefore, emotionally rewarding experiences are expected to strengthen both perceived functional value (because the platform is appraised as more beneficial in fulfilling entertainment needs) and satisfaction, and to promote approach intentions.

H3-1: Emotional experience positively influences users’ perceptions of functional value in OTT video platforms.

H3-2: Emotional experience positively influences users’ satisfaction in OTT video platforms.

H3-3: Emotional experience positively influences users’ usage intention in OTT video platforms.

Service quality experience captures system/service reliability and transmission quality (e.g., few crashes, rare content removal, smooth streaming). Service quality theory and IS success models suggest that reliable system quality is a foundational antecedent of perceived usefulness/value and user satisfaction [32,33]. However, motivation-hygiene logic implies that reliability can operate as a “hygiene factor” [42]: once it meets a basic threshold, incremental improvements may contribute more to value and satisfaction than to intention directly.

H4-1: Service quality experience positively influences users’ perceptions of functional value in OTT video platforms.

H4-2: Service quality experience positively influences users’ satisfaction in OTT video platforms.

H4-3: Service quality experience positively influences users’ usage intention in OTT video platforms.

Perceived functional value reflects the user’s cognitive judgment that the platform is practical and worth the cost/effort. Consumption value theory indicates that utility/value perceptions shape post-consumption evaluations [43,44], and expectancy-confirmation research similarly argues that higher perceived benefits increase satisfaction [39,40]. Whether functional value translates directly into intention is debated: some studies find a direct effect, while others show that value mainly operates through satisfaction in hedonic-dominant services. We therefore posit both relationships to be tested in the OTT setting.

H5-1: Functional value positively influences users’ satisfaction in OTT video platforms.

H5-2: Functional value positively influences users’ usage intention in OTT video platforms.

Satisfaction is a post-consumption affective evaluation that summarizes whether experience outcomes meet or exceed expectations [39]. In continuance and post-adoption research, satisfaction is among the most consistent predictors of continued usage intention [40], serving as a proximal driver of approach behavior.

H6: Satisfaction positively influences users’ usage intention in OTT video platforms.

## Research method

### Data source and questionnaire structure

This study is based entirely on secondary survey data obtained from the Survey Research Data Archive (SRDA), Academia Sinica, Taiwan. The present authors did not conduct the original survey or recruit participants directly; rather, this study reanalyzed an existing archived dataset for secondary research purposes. The archived dataset contained 239 valid cases. After excluding respondents who had never used OTT platforms, 230 cases were retained for analysis.

All data made available through SRDA are fully anonymized and contain no personally identifiable information. The present authors had no access to any individual-level identifiers during or after data collection. According to the archive documentation available to the authors, the original survey was conducted under relevant ethical procedures. Because the present study involved only secondary analysis of anonymized archived data and did not involve direct interaction with human participants, no additional ethics approval or informed consent was required for the current analysis under institutional research ethics guidelines.

According to the archive documentation, the original questionnaire had been developed through a context-adaptation process. The initial measurement items were derived from validated constructs in prior studies and were subsequently adapted to the OTT context. The archived documentation further indicates that the revised item pool was reviewed by three industry experts, three academic professors, and four highly involved users for content relevance, wording clarity, and contextual appropriateness.

All information reported below regarding questionnaire design, sample characteristics, and survey implementation is based on the archive documentation associated with the original dataset rather than on a new survey administered by the present authors.

The questionnaire was divided into five sections:

1. Demographics.
2. Screening questions, such as “Have you ever used OTT video platforms?”, “Which platform do you use most?”, and “How long have you been using OTT video platforms?”.
3. Experience-related items. According to the archived questionnaire, the original instrument covered multiple experience-related items; however, the present study retained only four experience constructs in the final model: convenience and fluency experience, interactive experience, emotional experience, and service quality experience.
4. Functional value.
5. Satisfaction and usage intention.

All constructs were measured using a 7-point Likert scale, ranging from 1 (“strongly disagree”) to 7 (“strongly agree”).

### Research Framework

**Fig 2:**
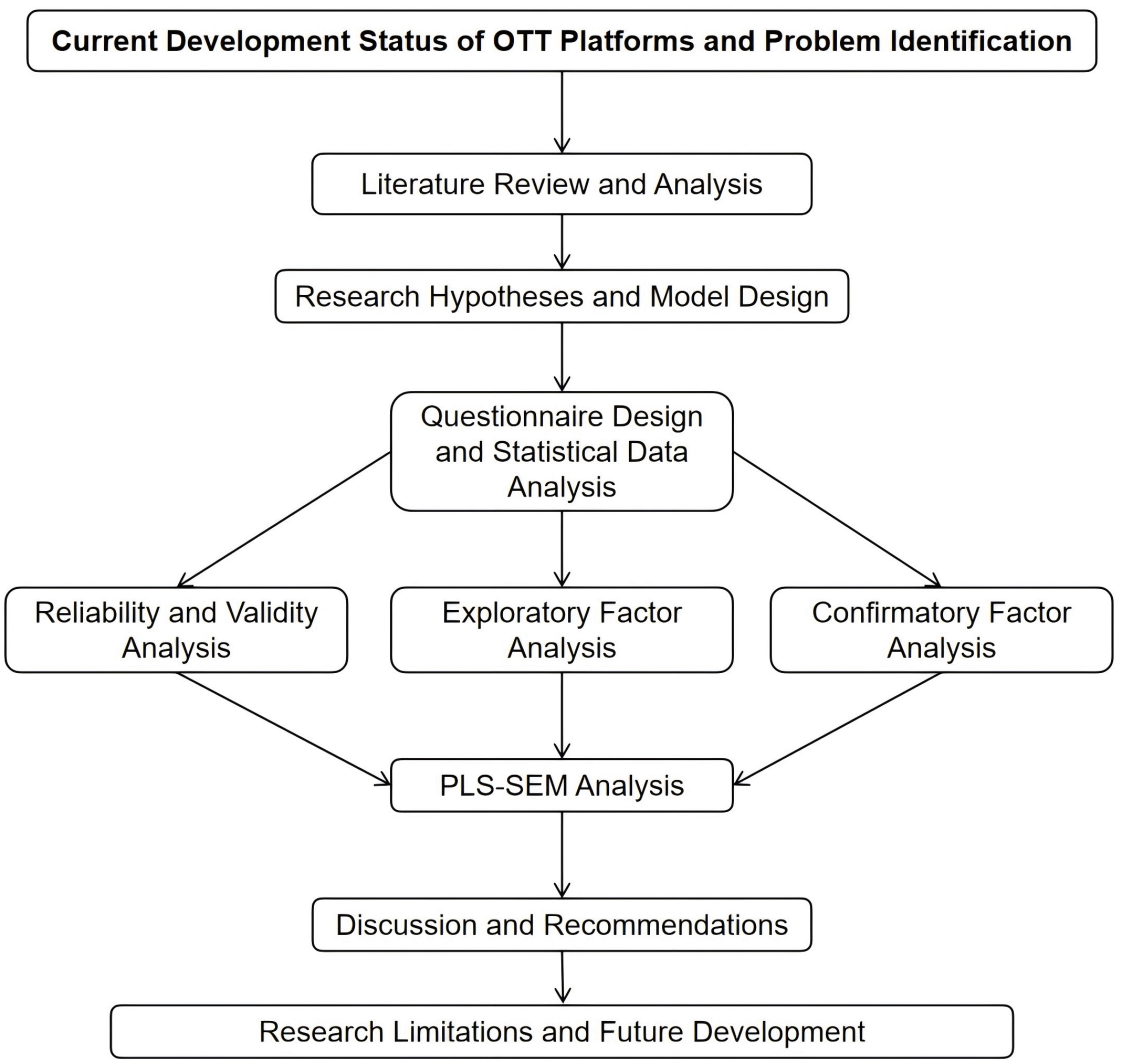
Research Framework.

Based on the proposed hypotheses and the adapted questionnaire constructs, a conceptual model of user experience, functional value, satisfaction, and usage intention in OTT video platforms was developed (see Fig 2).

### Data analysis

This study employed SPSS 20 and SmartPLS 3 statistical software for data analysis. Following the research objectives and hypotheses, we conducted descriptive statistical analysis, reliability and validity testing, confirmatory factor analysis, and structural model evaluation.

### SmartPLS modeling specifications and analysis

The study adopted partial least squares structural equation modeling (PLS-SEM) using SmartPLS 3 [45,46], and confirmatory factor analysis was conducted via the software. PLS-SEM is suitable for datasets that are non-normally distributed, as well as for relatively small sample sizes and complex models [47,48]. Since measurement items were based on perceptual ratings, the data often exhibited non-normal distribution [49]. Consistent with Lee et al. [50], the Kolmogorov–Smirnov test indicated p-values < 0.05, confirming the data’s non-normality and validating the application of PLS-SEM. Despite the relatively small sample size (230 valid responses), the dataset satisfied the minimum sample size requirement proposed by Soper [51].

### Assessment of the measurement model

Before evaluating the final measurement model, it is important to distinguish between the preliminary diagnostic analyses and the formal PLS-SEM assessment. Tables 1 and 2 report item-level descriptive diagnostics and exploratory factor analysis (EFA) results, which were used to examine data quality and the initial measurement structure. The formal assessment of the measurement model was then conducted in the PLS-SEM stage, and the reliability, convergent validity, and discriminant validity results reported in Tables 3–6 were used as the primary basis for judging the adequacy of the final measurement model.

**Table 1:** Item-level descriptive statistics and preliminary loading diagnostics.

Item	Question	Missing values	Mean	Std. Dev.	Skewness	t-test	Correlation	Factor Loading
Q1	The platform updates programs very quickly	0%	5.78	1.274	−1.413	7.272 ***	0.442	0.599
Q2	The platform streams programs smoothly	0%	5.75	1.104	−1.364	9.137 ***	0.564	0.717
Q3	The platform offers high-quality video resolution	0%	5.57	1.164	−0.787	12.695 ***	0.632	0.687
Q4	The platform’s user interface is clear and easy to understand	0%	5.93	1.028	−1.181	10.172 ***	0.641	0.86
Q5	The overall visual presentation of the platform is attractive	0%	5.34	1.193	−0.582	11.477 ***	0.622	0.722
Q6	The platform is convenient to use across multiple devices	0%	5.97	1.15	−1.574	7.779 ***	0.513	0.704
Q7	I enjoy chatting with others on this platform	0%	2.65	1.478	0.78	2.685 ***	0.118	0.784
Q8	I like to freely express my opinions on this platform	0%	3.24	1.653	0.315	6.114 ***	0.33	0.879
Q9	When my opinions are liked or shared on this platform, I feel recognized	0%	4.23	1.475	−0.198	5.676 ***	0.308	0.663
Q10	Compared to other video services, I find this platform’s content joyful	0%	5.59	1.113	−0.811	12.631 ***	0.661	0.941
Q11	Compared to other video services, I feel happy with this platform’s content	0%	5.6	1.076	−0.692	12.568 ***	0.68	0.941
Q12	The platform’s system service quality is stable (e.g., few crashes, rare content removal)	0%	5.25	1.269	−0.791	8.377 ***	0.485	0.815
Q13	The platform’s transmission quality is smooth (e.g., no disconnections or streaming delays)	0%	5.35	1.257	−0.813	10.543 ***	0.575	0.815
Q14	Compared to other video services, I feel this platform is good value for money	0%	5.55	1.179	−0.64	11.793 ***	0.569	0.776
Q15	Compared to other video services, I find this platform practical for me	0%	5.76	1.108	−1.152	11.762 ***	0.674	0.9
Q16	Even if this platform’s market share is low, I still find it worthwhile	0%	5.39	1.299	−0.63	7.761 ***	0.431	0.515
Q17	Compared to other video services, I feel satisfied with this platform’s content	0%	5.61	1.059	−0.83	13.729 ***	0.698	0.864
Q18	Overall, I am satisfied with this platform’s content	0%	5.76	1.006	−1.137	12.038 ***	0.74	0.864
Q19	I intend to increase my frequency of using this platform in the next six months	0%	5.14	1.183	−0.38	10.883 ***	0.599	0.79
Q20	I intend to increase my use of this platform to replace other media (e.g., TV, movies)	0%	4.99	1.323	−0.615	8.780 ***	0.515	0.755
Q21	I intend to recommend this platform to others in the next six months	0%	5.12	1.2	−0.43	10.082 ***	0.569	0.857

**Table 2:** Factor Analysis.

Experience					
**Factor**	**Item**	**Factor 1**	**Factor 2**	**Factor 3**	**Factor 4**
Convenience and Fluency Experience	Q6 The platform is convenient to use across multiple devices	0.819			
	Q4 The platform’s user interface is clear and easy to understand	0.756			
	Q5 The platform’s overall visual presentation is attractive	0.734			
	Q1 The platform updates programs very quickly	0.677			
	Q3 The platform offers high-quality video resolution	0.671			
	Q2 The platform streams programs smoothly	0.653			
Interactive Experience	Q8 I like to freely express my opinions on this platform		0.882		
	Q7 I enjoy chatting with others on this platform		0.842		
	Q9 When my opinions are liked or shared on this platform, I feel a sense of recognition		0.765		
Emotional Experience	Q10 Compared to other video services, I feel joyful with this platform’s content			0.839	
	Q11 Compared to other video services, I feel happy with this platform’s content			0.821	
Service Quality Experience	Q12 The platform’s system service quality is stable (e.g., few crashes, rare content removal)				0.88
	Q13 The platform’s transmission service quality is smooth (e.g., no disconnection or delay)				0.869
Explained Variance (%)		39.728	17.385	9.324	7.461
Cumulative Variance Explained (%)		39.728	57.113	66.437	73.897
Cronbach’s α	0.824	KMO Value	0.814	p-value	0
**Perceived Functional Value**					
**Factor**	**Item**	**Factor 1**			
Functional Value Perception	Q14 Compared with other video services, using this platform makes me feel it is worth the value	0.89			
	Q15 Compared with other video services, I think using this platform is practical	0.863			
	Q16 Even if this platform’s market share is low, I still find it worthwhile	0.719			
Explained Variance (%)		68.429			
Cumulative Variance Explained (%)		68.429			
Cronbach’s α	0.757	KMO Value	0.636	p-value	0
**User Satisfaction**					
**Factor**	**Item**	**Factor 1**			
Satisfaction	Q17 Compared with other video services, I feel satisfied with the program content of this platform	0.935			
	Q18 Overall, I am satisfied with the program content of this platform	0.935			
Explained Variance (%)		87.39			
Cumulative Variance Explained (%)		87.39			
Cronbach’s α	0.855	KMO Value	0.5	p-value	0
**Usage Intention**					
**Factor**	**Item**	**Factor 1**			
Usage Intention	Q19 In the next six months, I will increase my frequency (times) of using this platform	0.892			
	Q20 In the next six months, I will increase my use of this platform to replace other media (e.g., TV, film)	0.869			
	Q21 In the next six months, I will recommend this platform to other people	0.855			
Explained Variance (%)		76.022			
Cumulative Variance Explained (%)		76.022			
Cronbach’s α	0.84	KMO Value	0.721	p-value	0

**Table 3:** Factor loadings, average variance extracted, internal consistency, R², and composite reliability.

	Constructs	Interactive Experience	Usage Intention	Convenience and Fluency Experience	Perceived Functional Value	Emotional Experience	Service Quality Experience	Satisfaction
Factor Loadings	1	0.731	0.879	0.682	0.870	0.970	0.926	0.936
	2	0.903	0.844	0.773	0.911	0.972	0.898	0.934
	3	0.860	0.892	0.771	0.677			
	4			0.865				
	5			0.773				
	6			0.747				
	AVE	0.697	0.760	0.594	0.682	0.943	0.831	0.874
	Cronbach’s Alpha	0.813	0.842	0.862	0.765	0.939	0.798	0.856
	R²		0.339		0.522			0.760
	Composite reliability	0.872	0.905	0.897	0.864	0.971	0.908	0.933

(Note: AVE > 0.5; CR > 0.6; Cronbach’s α > 0.7; Factor loading > 0.5)

**Table 4:** Discriminant validity analysis.

	Interactive Experience	Usage Intention	Convenience and Fluency Experience	Functional Value Perception	Emotional Experience	Service Quality Experience	Satisfaction
Interactive Experience	0.835						
Usage Intention	0.228**	0.872					
Convenience and Fluency Experience	0.114**	0.484**	0.77				
Functional Value Perception	0.081**	0.462**	0.586**	0.826			
Emotional Experience	0.044**	0.474**	0.576**	0.666**	0.971		
Service Quality Experience	0.098**	0.307**	0.519**	0.51**	0.502**	0.912	
Satisfaction	0.095**	0.512**	0.655**	0.726**	0.816**	0.576**	0.935

**Table 5:** HTMT value.

	Interactive Experience	Usage Intention	Convenience and Fluency Experience	Functional Value Perception	Emotional Experience	Service Quality Experience	Satisfaction
Interactive Experience							
Usage Intention	0.248						
Convenience and Fluency Experience	0.185	0.559					
Functional Value Perception	0.147	0.582	0.693				
Emotional Experience	0.113	0.532	0.636	0.768			
Service Quality Experience	0.129	0.372	0.624	0.629	0.579		
Satisfaction	0.122	0.601	0.76	0.877	0.909	0.693	

**Table 6:** VIF.

VIF value			
Q1	1.597	Q11	1.79
Q2	1.906	Q12	2.126
Q3	1.791	Q13	1.293
Q4	2.566	Q14	4.641
Q5	1.97	Q15	4.641
Q6	1.881	Q16	2.269
Q7	2.307	Q17	2.269
Q8	2.652	Q18	1.79
Q9	1.468	Q19	2.005
Q10	1.981	Q20	1.868
		Q21	2.226

(Note: VIF < 10).

To evaluate the measurement model, both reliability and validity were examined [52]. In the preliminary diagnostic stage, factor loadings were examined to assess the initial measurement structure. The final evaluation of retained constructs was based on the PLS-SEM results reported in Tables 3–6. Internal consistency reliability was assessed using Cronbach’s alpha (α > 0.7) and composite reliability (CR > 0.6) [53]. When Cronbach’s alpha fell below 0.7, deletion of items was considered to ensure internal consistency [54].

Convergent validity was verified by ensuring that the average variance extracted (AVE) exceeded 0.50 and that indicator loadings were acceptable [55, 56]. Discriminant validity was assessed using both the Fornell–Larcker criterion and the heterotrait-monotrait ratio (HTMT) [57]. Although the Fornell–Larcker criterion was satisfied, the HTMT value between Emotional Experience and Satisfaction reached 0.909. Therefore, discriminant validity was regarded as acceptable but borderline and was interpreted with caution.

### Assessment of the Structural Model

Following Hair Jr. et al. [58], collinearity was tested using VIF values, with thresholds of VIF < 10 indicating acceptable collinearity. For inner model evaluation, two primary criteria were applied: (1) R² values must exceed 0.1 to demonstrate explanatory power, and (2) t-values of path coefficients must reach significance to confirm hypothesized relationships.

## Results

### Descriptive statistics

This study analyzed secondary survey data obtained from an archived SRDA dataset on emerging media platform adoption. The archived dataset contained 239 valid cases, and after excluding respondents who had never used OTT platforms, 230 cases were retained for analysis.

The final sample consisted of 230 respondents and was concentrated among young and highly educated users. Females accounted for 71.7% of the participants, indicating a gender imbalance that may reflect higher engagement or interest among female users in the research context. In terms of age, most respondents were between 21 and 30 years old (44.8%), followed by those aged ≤20 (38.7%), suggesting that the sample primarily represents younger populations, particularly students or early-career individuals. Regarding education level, participants were predominantly well educated, with 74.8% holding a bachelor’s degree and 11.3% holding a graduate degree.

Overall, the demographic profile indicates that the findings primarily reflect the perceptions of young and highly educated users. Although this composition is appropriate for research on digital platforms, it may limit the generalizability of the findings to older or less-educated populations.

### Assessing the measurement model

The survey was designed to capture users’ experiential perceptions of OTT platforms. Prior to structural analysis, data quality was examined through seven diagnostic indicators, including missing values, mean, standard deviation, skewness, t-tests, correlations, and factor loadings. All results fell within acceptable ranges (see Table 1).

Subsequently, exploratory factor analysis (EFA) was conducted to validate construct naming and measurement structure, with details summarized in Table 2.

Given that the data were collected using a single questionnaire at one point in time, the potential for common method bias (CMB) was examined. Following Harman’s single-factor test, all measurement items were entered into an unrotated exploratory factor analysis. The first factor accounted for 39.728% of the total variance, which is below the commonly used threshold of 50%. In addition, all VIF values were below the recommended threshold, suggesting that severe multicollinearity is unlikely. However, because the study relied on cross-sectional self-reported data, common method bias cannot be completely ruled out. Therefore, the findings should be interpreted with caution, and future research is encouraged to use multi-source data, longitudinal designs, or behavioral indicators.

### Measurement Model Evaluation (PLS-SEM Results)

This study employed SmartPLS 3 to conduct Partial Least Squares Structural Equation Modeling (PLS-SEM) [45,46], and confirmatory factor analysis (CFA) was performed using the same software. PLS-SEM is particularly suitable for datasets that deviate from normal distribution, as well as for studies involving small sample sizes and relatively complex models [47].

Since the measurement items were obtained through perceived rating scales, the data could not be assumed to follow a normal distribution [49]. Following Lee et al. [50], the Kolmogorov–Smirnov normality test was applied, and results showed all p-values < 0.05, indicating that the dataset is non-normally distributed. Therefore, the adoption of the PLS-SEM approach is justified.

Furthermore, based on the PLS-SEM results reported in Tables 3–6, the retained constructs satisfied the required thresholds for reliability, convergent validity, and discriminant validity, confirming the adequacy of the final measurement model.

Although the reliability and validity statistics in Tables 3–6 meet conventional PLS-SEM benchmarks, we provide additional interpretation for indicators that are relatively close to critical thresholds. First, internal consistency is acceptable across constructs, with Cronbach’s alpha ranging from 0.765 to 0.939 and composite reliability (CR) ranging from 0.864 to 0.971 (Table 3), both exceeding the recommended 0.70 criterion. Importantly, the lowest alpha appears for perceived functional value (α = 0.765), which is still above the threshold and accompanied by strong CR (0.864), suggesting adequate reliability despite being comparatively lower than other constructs. Second, convergent validity is supported because all AVE values exceed 0.50 (0.594–0.943). The AVE for Convenience and Fluency Experience is the lowest (AVE = 0.594) and therefore closest to the cut-off, which is consistent with the presence of several moderate indicator loadings (e.g., one item at 0.682 and others around 0.747–0.773). Nevertheless, these loadings remain above 0.50 and, together with satisfactory CR and AVE, indicate that the construct retains acceptable convergence while preserving content coverage. Third, discriminant validity was verified using both the Fornell-Larcker criterion (Table 4) and HTMT (Table 5). Although the Fornell–Larcker criterion was satisfied, discriminant validity should be interpreted with caution because the HTMT value between Emotional Experience and Satisfaction reached 0.909, indicating potential conceptual overlap. Therefore, discriminant validity can be regarded as acceptable but borderline rather than unequivocally established. Finally, potential multicollinearity was examined using VIF, and all indicators were below 10 (Table 6), suggesting that collinearity is unlikely to bias the estimates. Overall, the measurement model demonstrates adequate psychometric quality; however, constructs with comparatively lower AVE or moderate loadings should be interpreted with appropriate caution, and future research is encouraged to refine items for the Convenience and Fluency Experience and to replicate the measurement model in more heterogeneous samples.

### Examining the structural model

To assess the stability of the model, bootstrapping with 5,000 resamples was performed using SmartPLS. According to [59], the R² coefficient is an appropriate measure for evaluating the explanatory power of the structural model. In this study, the R² values were 0.339 for usage intention, 0.522 for perceived functional value, and 0.760 for satisfaction (see Table 7). These results indicate that the structural model provides acceptable explanatory power for the endogenous constructs. Predictive relevance was further assessed using Stone–Geisser’s Q² statistic, as reported in Table 7.

**Table 7:** Predictive relevance of the endogenous constructs.

	R^2^	Q^2^
Usage Intention	0.339	0.240
Functional Value Perception	0.522	0.339
Satisfaction	0.760	0.648

### Predictive relevance

In addition to R², this study also employed Stone–Geisser’s Q² statistic to evaluate the predictive relevance of the endogenous constructs. Following Wong et al. [60], Q² > 0.35 indicates high predictive relevance, 0.35 > Q² > 0.15 indicates moderate predictive relevance, 0.15 > Q² > 0.05 indicates low predictive relevance, and Q² < 0.05 suggests no predictive relevance. The results confirmed that the model has acceptable levels of predictive relevance for the endogenous constructs (see Table 7).

As shown in Table 7, the Q² statistics indicate that the model provides acceptable levels of predictive relevance. Specifically, the Q² value for usage intention was 0.24, which falls within the range of moderate predictive relevance (0.15 < Q² < 0.35). The Q² value for functional value perception was 0.339, also reflecting moderate predictive relevance, while the Q² value for satisfaction reached 0.648, exceeding the 0.35 threshold and thus demonstrating high predictive relevance. These results suggest that the endogenous constructs in the model exhibit satisfactory predictive capabilities.

Traditionally, PLS-SEM has been regarded as a prediction-oriented method and has lacked global model fit indices. The introduction of the Standardized Root Mean Square Residual (SRMR) addresses this limitation by providing a measure of overall model fit [61,62]. According to Hu and Bentler [62], an SRMR value below 0.08 is generally considered indicative of good model fit. In this study, the estimated SRMR was 0.075, thereby confirming that the structural model demonstrates an acceptable level of overall fit.

### Mediation Effect Testing

The mediation effects in this study were examined following the criteria proposed by [59]. Specifically, when the Variance Accounted For (VAF) value is greater than 80%, it indicates full mediation; when 20% < VAF < 80%, it indicates partial mediation; and when VAF < 20%, it indicates no mediation.

**Fig 3:**
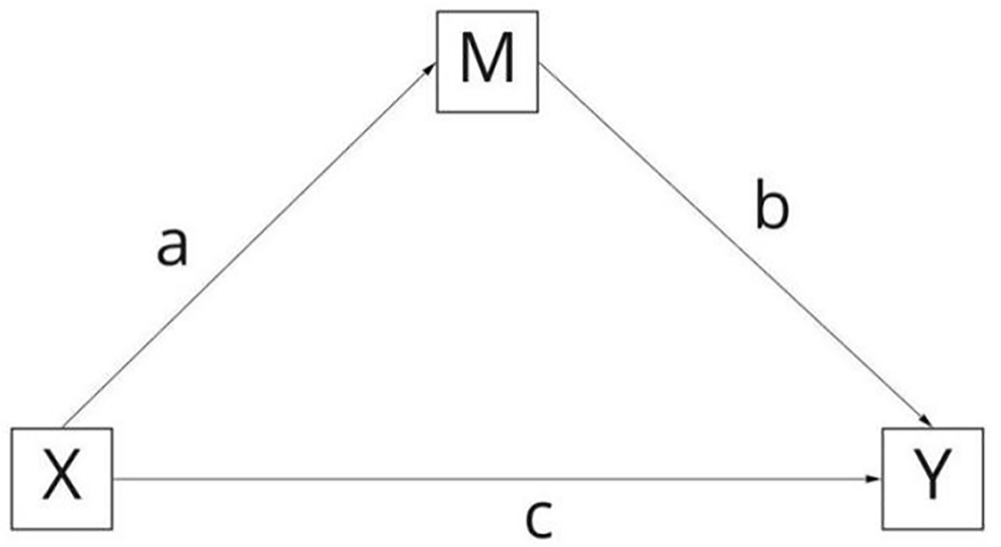
Mediation Effect Model.

In the context of PLS-SEM, researchers commonly assess the strength of mediation by calculating the ratio of the indirect effect to the total effect. This ratio, referred to as the Variance Accounted For (VAF), provides a clear measure of the extent to which the mediating variable transmits the effect of the independent variable to the dependent variable. The specific operations are as follows (see Fig 3 for the mediation model):

Step 1: Test whether the independent variable (X) significantly predicts the dependent variable (Y) (path coefficient c).

Step 2: Test whether X significantly predicts the mediator (M) (path coefficient a), and whether M significantly predicts Y (path coefficient b).

Step 3: Run the full mediation model, simultaneously verifying the significance of coefficients (a, b, and c), and calculate the explained variance using the Variance Accounted For (VAF).

The formula for VAF is expressed as:


$$\begin{array}{c} \text{VAF}=\frac{\text{a}\times \text{b}}{(\text{a}\times \text{b})+\text{c}} \end{array}$$

(Note: a, b, and c represent path coefficients).

Based on Table 8, satisfaction partially mediated the relationship between emotional experience and usage intention. Functional value perception also showed partial mediation in the relationships from convenience and fluency experience and service quality experience to satisfaction. By contrast, the indirect path from emotional experience to satisfaction via functional value perception showed only a limited mediation ratio (VAF = 16.69%) and was therefore not interpreted as substantive mediation. These findings are summarized in Table 8.

**Table 8:** Mediation Effects.

Paths	Indirect Effect	Total Effect	VAF	Mediation Type
Emotional Experience → Satisfaction → Usage Intention	0.118	0.304	38.82%	Partial Mediation
Convenience and Fluency Experience → Functional Value Perception → Satisfaction	0.056	0.221	24.34%	Partial Mediation
Service Quality Experience → Functional Value Perception → Satisfaction	0.036	0.152	23.68%	Partial Mediation
Emotional Experience → Functional Value Perception → Satisfaction	0.102	0.611	16.69%	No Mediation

## Discussion

### Direct Effects of UX Attributes on Evaluations and Behavioral Intentions

**Fig 4:**
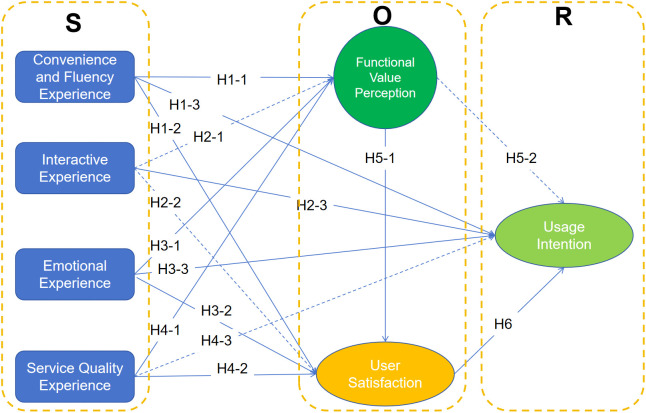
OTT User Experience Model.

The empirical results indicate that UX attributes influence OTT users’ evaluations and behavioral intentions in differentiated ways, rather than uniformly supporting all hypothesized relationships.(see Table 9 and see Fig 4).

**Table 9:** Results of the structural model.

Hypotheses	Paths	Path coefficients (β)	T-values	P Values	Remarks
**H1-1**	Convenience and Fluency Experience → Functional Value Perception	0.246	3.637	p < .001	Supported
**H1-2**	Convenience and Fluency Experience → Satisfaction	0.221	4.408	p < .001	Supported
**H1-3**	Convenience and Fluency Experience → Usage Intention	0.298	4.418	p < .001	Supported
**H2-1**	Interactive Experience → Functional Value Perception	0.018	0.305	0.76	Unsupported
**H2-2**	Interactive Experience → Satisfaction	0.028	0.74	0.459	Unsupported
**H2-3**	Interactive Experience → Usage Intention	0.182	2.39	0.017	Supported
**H3-1**	Emotional Experience → Functional Value Perception	0.445	6.433	p < .001	Supported
**H3-2**	Emotional Experience → Satisfaction	0.611	10.534	p < .001	Supported
**H3-3**	Emotional Experience → Usage Intention	0.304	4.306	p < .001	Supported
**H4-1**	Service Quality Experience → Functional Value Perception	0.157	2.486	0.013	Supported
**H4-2**	Service Quality Experience → Satisfaction	0.152	2.611	0.009	Supported
**H4-3**	Service Quality Experience → Usage Intention	−0.018	0.262	0.794	Unsupported
**H5-1**	Functional Value Perception → Satisfaction	0.229	4.256	p < .001	Supported
**H5-2**	Functional Value Perception → Usage Intention	0.053	1.725	0.085	Unsupported
**H6**	Satisfaction → Usage Intention	0.232	2.024	0.043	Supported

First, convenience and fluency experience had significant positive effects on functional value perception (H1-1), satisfaction (H1-2), and usage intention (H1-3). This result is consistent with prior studies emphasizing usability and effort reduction as key antecedents of perceived usefulness and continuance in digital services [37–40]. The magnitude of the path coefficients suggests that a smooth viewing process functions as a fundamental condition that supports value appraisal and satisfaction while also contributing to intention. In practical terms, improvements in playback stability, interface clarity, and cross-device accessibility appear to contribute to retention over time rather than acting as decisive drivers on their own.

Second, Interactive Experience significantly predicted usage intention (H2-3) but did not significantly affect functional value perception (H2-1) or satisfaction (H2-2). This pattern differs from research that positions interactivity as strengthening overall evaluation and instead suggests that, in OTT environments, interaction operates primarily through engagement and social participation [35,36]. Users may continue using a platform because interaction features sustain involvement and connection, even when these features do not substantially alter perceptions of usefulness or overall satisfaction. This finding helps account for inconsistent conclusions in earlier studies and indicates that interactivity may function more as an engagement-oriented mechanism than as a direct source of utilitarian value.

Third, emotional experience showed the strongest and most consistent effects across the model. It significantly predicted functional value perception (H3-1), satisfaction (H3-2), and usage intention (H3-3), and also influenced intention indirectly through satisfaction. This result supports cognitive-appraisal perspectives that emphasize the role of affective responses in shaping evaluative judgments and behavioral tendencies [34,41]. Emotional reactions therefore appear to be central to how users interpret value and decide whether to continue using OTT services.

Fourth, service quality experience significantly affected functional value perception (H4-1) and satisfaction (H4-2) but did not directly influence usage intention (H4-3). This pattern suggests that system reliability and stable performance function as baseline conditions. They contribute to positive evaluations but do not necessarily motivate continued use once basic expectations are satisfied [32,33]. The results further indicate that users translate reliable performance into perceptions of usefulness, which subsequently shape their overall evaluation.

Fifth, functional value perception significantly enhanced satisfaction (H5-1) but did not directly predict usage intention (H5-2). This finding implies that utilitarian evaluations alone may not be sufficient to drive continued use in a hedonic-oriented environment such as OTT video. Instead, perceived value appears to operate primarily by strengthening satisfaction, which then influences behavioral intention [44,43]. Functional value thus plays a supportive role in the evaluative process rather than acting as a direct behavioral trigger.

Finally, satisfaction had a significant positive effect on usage intention (H6), confirming its role as a proximal determinant of continued platform use [39,40]. As a post-consumption evaluation, satisfaction integrates both cognitive assessments and affective responses and translates them into behavioral tendencies.

Overall, these findings extend the application of the S-O-R framework in the OTT context by showing that UX dimensions do not operate through a single mechanism. Convenience and fluency influence both evaluative processes and usage intention directly, whereas service quality mainly shapes evaluative responses. Emotional experience shows the strongest effects on satisfaction and usage intention, and interaction is directly associated with usage intention rather than with functional value or satisfaction. This indicates that, in OTT environments, stimulus effects are differentiated rather than uniform, and that cognitive and affective organism states are activated selectively across UX dimensions. This more explicit pattern helps explain why previous studies have reported inconsistent findings regarding interactivity and utilitarian value in entertainment-oriented digital services.

### Mediation effects and internal mechanisms

The mediation analysis further clarifies how different UX attributes operate through distinct internal mechanisms in the OTT context. Rather than indicating a uniform mediation pattern across all paths, the results suggest that indirect effects are selective and depend on the nature of the specific experience attribute.

First, satisfaction mediated the relationship between emotional experience and usage intention. This result indicates that emotional responses promote continued platform use not only through a direct motivational route but also through a post-consumption evaluative route. In other words, emotionally engaging content appears to strengthen users’ intention to continue using the platform both because it is directly appealing and because it enhances overall satisfaction.

Second, perceived functional value mediated the relationship between convenience and fluency experience and satisfaction. A seamless viewing process, intuitive interface, and smooth cross-device access seem to contribute to satisfaction partly because users interpret these characteristics as signs of practical value and efficiency. At the same time, the continued significance of the direct path suggests that convenience and fluency also shape satisfaction more immediately at the experiential level.

Third, perceived functional value also mediated the relationship between service quality experience and satisfaction. Stable transmission and reliable system performance appear to enhance satisfaction partly by reinforcing users’ judgments that the platform is useful and worthwhile. This finding supports the view that service quality contributes to positive evaluations not only as a background condition, but also through users’ cognitive appraisal of platform utility.

By contrast, there is weaker evidence that perceived functional value substantially mediates the relationship between emotional experience and satisfaction. This suggests that emotional experience influences satisfaction primarily through a direct affective route rather than through an extended cognitive evaluation process. In the OTT context, positive emotional responses may therefore function as a relatively immediate basis for satisfaction.

Taken together, these findings point to a differentiated S-O-R mechanism. Emotional experience primarily operates through affective evaluation, whereas convenience/fluency and service quality are linked to satisfaction partly through cognitive value appraisal. The internal mechanism is therefore not uniform across UX dimensions; instead, different experiential cues activate different evaluative pathways before shaping users’ intentions and post-consumption judgments.

## Conclusion

This study examined how key UX attributes of OTT platforms are associated with users’ evaluations and continuance intentions under the S-O-R framework, and identified differentiated patterns linking usability, emotional experience, interaction, and service quality to behavioral outcomes. The results indicate that emotional experience and satisfaction play central roles in shaping usage intention. Convenience and fluency contribute to value appraisal, satisfaction, and usage intention, whereas service quality mainly supports value appraisal and satisfaction. Interaction, in contrast, is directly associated with continued use, while showing no significant effects on perceived functional value or satisfaction. This pattern suggests that its role may be more closely related to engagement than to evaluative judgment.

Beyond these theoretical implications, the findings provide several concrete managerial insights for OTT platform operators.

First, improving convenience and fluency should remain a basic operational priority. Stable playback, intuitive interfaces, fast loading speeds, and seamless cross-device access were shown to enhance both satisfaction and intention. These elements may not generate strong differentiation individually, but deficiencies in them can quickly reduce user retention. Platform managers should therefore treat usability optimization as a continuous, infrastructure-level task rather than a one-time improvement.

Second, emotional experience emerged as the most influential factor across the model. This suggests that content strategy should focus not only on availability but also on emotional resonance. Platforms can strengthen user retention by prioritizing emotionally engaging content, improving recommendation systems to match users’ affective preferences, and curating thematic viewing experiences (e.g., mood-based playlists, scenario-driven content bundles). Compared with purely technical improvements, investments in emotionally appealing content are more likely to translate into sustained usage.

Third, interaction features should be positioned as engagement tools rather than as mechanisms for improving perceived usefulness. Social commenting, sharing, and feedback functions appear to motivate continued use by strengthening involvement and community participation. Platform managers may therefore focus on improving participation design—such as real-time interaction, creator–viewer communication, and community-based content activities—rather than expecting interaction alone to increase satisfaction.

Fourth, service quality should be maintained as a core reliability condition. The results indicate that stable transmission, minimal technical failures, and consistent content availability contribute to users’ evaluative responses, especially satisfaction, but do not show a significant direct effect on usage intention in the present model. Management efforts should therefore prioritize preventing service disruptions and maintaining consistency rather than relying on technical upgrades alone to attract users.

Fifth, functional value is positively associated with satisfaction but does not show a significant direct effect on usage intention. Its role therefore appears to be more closely tied to evaluative processes than to a direct behavioral pathway. This indicates that practical features, such as search efficiency, content organization, and subscription value should be designed to enhance the overall experience rather than treated as standalone drivers of retention. Managers should integrate functional improvements with user-experience design, ensuring that usability enhancements translate into perceived enjoyment and satisfaction.

Overall, the findings suggest a differentiated management logic: usability and service reliability should be maintained as foundational conditions, emotional engagement should be strengthened to enhance retention, interaction features should be used to support participation, and functional improvements should be aligned with user satisfaction and perceived value. Such a differentiated strategy may be more effective than uniformly investing in all UX dimensions, because different attributes influence usage intention through distinct mechanisms. More broadly, the findings highlight the importance of user-centered design in OTT platforms by showing that different experiential attributes contribute to continued use through different evaluative pathways.

### Limitations and future research

This study has several limitations that should be considered when interpreting the findings. First, the use of cross-sectional survey data limits the ability to draw causal inferences, as user perceptions and intentions may change over time. Second, the analysis treated OTT usage in a general sense without distinguishing between platform types, content genres, or user motivations, which may influence evaluation mechanisms. Third, the measurement of Interactive Experience mainly focused on participation and feedback features and did not fully capture other forms of interaction such as algorithmic personalization or co-creation. In addition, the study did not incorporate potential moderating variables, including individual media habits or hedonic–utilitarian orientations, which may explain the variability in some relationships. Finally, the reliance on self-reported perceptions and intentions may not fully reflect actual usage behavior.

Future research could address these limitations by employing longitudinal or experimental designs to better examine causal relationships and dynamic changes in user evaluations. Further studies may also differentiate between platform contexts, content types, and user segments to explore boundary conditions of UX effects. Expanding the conceptualization of interaction and incorporating behavioral data—such as viewing records or platform analytics—would provide a more comprehensive understanding of user engagement. These directions may help clarify how different UX attributes influence continuance mechanisms in OTT environments and strengthen the explanatory power of S-O-R–based models.
